# Editorial: New ideas in language sciences: linguistics

**DOI:** 10.3389/fpsyg.2023.1289877

**Published:** 2023-09-21

**Authors:** Rebecca Carroll, Mile Vukovic´, Moreno I. Coco, David Townsend

**Affiliations:** ^1^Department of English and American Studies, Technical University of Braunschweig, Braunschweig, Germany; ^2^Department of Special Education and Rehabilitation, University of Belgrade, Belgrade, Serbia; ^3^Department of Psychology, Sapienza University of Rome, Rome, Italy; ^4^Department of Psychology, Montclair State University, Montclair, NJ, United States

**Keywords:** language, linguistics, empirical research, theory-building, diversity, new ideas

New ideas arising from sub-disciplines of linguistics and disciplines outside linguistics have advanced the field on core topics such as theory-building, comprehension, production, and acquisition of language. Advances in computational linguistics made it possible to challenge traditional approaches based on grammatical theories by providing powerful stochastic or Bayesian models of language. Co-registration between behavioral and neural responses has contributed to shed light on the mechanisms of language processing. Additionally, the intersection between linguistics and other disciplines extends to comparisons of multilingualism as well as (non-)degenerative language disorders. Such research has demonstrated that including diverse populations in linguistic research can bolster our understanding of language. Finally, we assert that language is quintessential to social interaction, which emphasizes the importance of ecological and usage-based approaches.

Despite the important advances achieved by interdisciplinary work, the field still needs a comprehensive overarching framework that may account for the various cognitive and social mechanisms concurrently at play, such that we could aspire to truly explain “how language works.” Our goal with this Research Topic (*New ideas in language sciences: linguistics*) was to encourage submissions exploring linguistics across different perspectives and domains of knowledge. The seven papers that were included in this Research Topic helped us move toward this ambitious goal. They span several linguistic subfields, examine second language processes, and empirically cover the diversity of languages.

Through a judgment task on native Mandarin Chinese speakers, Wu challenges the widely held view that Mandarin allows only surface scope, unlike English which allows inverse scope. The study identified lexical and syntactic conditions that influence scope interpretations. It extends the use of advanced statistical methods in experimental syntax to non-Western languages. Similarly, Reimer and Smolka challenge existing theoretical accounts of idiom processing and thus serve as a base for future developments in this field. By experimentally dissociating effects of argument structure and argument and adjacency, they found compelling evidence that idioms may keep or change their figurative meaning based on a combination of grammatical voice and the adjacency of the verb to its critical arguments. Their findings seem to challenge existing theoretical accounts of idiom processing and thus serve as a base for future developments in this field. In addition to the continuing trend toward experimental investigations, we also see advances to move from carefully designed lab-based experiments to linguistic fieldwork. Butler examined the role of conceptual number in the comprehension of sentences in Yucatec Maya, a language with optional morphological marking of number. The results suggest cross-linguistic differences in access to morphological vs. conceptual information during comprehension. This study demonstrates that diversifying the languages that language scientists examine can reveal the flexibility of the language processing system.

Another important aspect of variation in the language sciences, beyond linguistic differences, pertains to different populations. Adding to the pool of existing data for multilingual speakers, albeit contributing to very different types of data and languages involved, two papers focus on English as a foreign language. Schlechtweg et al. showed how sociophonetic variation in the L1 plays a role in L2 acquisition. Using advanced state-of-the-art statistics, they found that the degree to which two vowels are merged or distinct in someone's L1, such as /e:/ and /ε:/ in German affects the success of phoneme distinction in the L2 (here: L2 English /ε/-/æ/). Their data underline the need for models of L2 acquisition and bilingual processing to extend to allow for individual sociophonetic variation on the L1 level. Çiftlikli and Demirel focus on the development of pragmatic competence and reading comprehension in the L2. Their data by university students seem to suggest that reading comprehension skills positively correlate with the comprehension of conversational implicatures in English as a foreign language. Understanding what is said and why it is said may help learners maintain effective communication and may boost their achievements in reading comprehension. The authors point out potential pedagogical implications of teaching language learners about conversational implicatures. A second aspect pertaining to variation in language population is hearing impairment. Zhang et al. investigated the development of speech-reading skills in Chinese speakers with hearing impairment. The results differ from the pattern found in English but are like the pattern found in French. Their study is notable for studying language skills in hearing-impaired populations and for highlighting the importance of studying diverse languages.

A continuing trend toward empirically testing “how language works” outside the traditional healthy young adult and native speaker of English populations is evident. Although neurolinguistic approaches based on experimental data were not submitted to this Research Topic, the perspective study by Alekseeva et al. argues for neurocognitive experimentation in linguistic research to provide “reality” and ways to falsify linguistic concepts that have canonically been assumed to exist on a purely theoretical, or logical, basis. The authors build the case around the inflectional zero-morpheme, which is a null constituent found across different languages. It is hard to be empirically grounded as, by definition, it does not have any overt physical and measurable expression. Besides reviewing different theoretical viewpoints about the zero-morpheme, which are motivated by idiosyncrasies observed across languages, the authors propose an interesting experimental electrophysiological research program that could help shed light on the neurocognitive reality of this specific morphological aspect of language.

We conclude that studying diverse languages across populations reveals that the human language processor is flexible. Even if grammatical knowledge may govern human language processing through universal constraints, it seems that it readily adapts to language-specific characteristics. All in all, it emerges that research in linguistics should span various languages and diverse populations such that we could establish, with greater certainty, possibly universal processing constraints while defining the limits of linguistic flexibility.

## Author contributions

RC: Writing—original draft, Writing—review and editing. MV: Writing—review and editing. MC: Writing—review and editing. DT: Writing—review and editing.

